# Concentration Dependent Asymmetric Synergy in SDS–DDAO Mixed Surfactant Micelles

**DOI:** 10.1021/acs.langmuir.3c03900

**Published:** 2024-03-27

**Authors:** Luis M. G. Torquato, Gunjan Tyagi, William N. Sharratt, Zain Ahmad, Najet Mahmoudi, Jérémie Gummel, Eric S. J. Robles, João T. Cabral

**Affiliations:** † Department of Chemical Engineering, Imperial College London, London SW7 2AZ, U.K.; ‡ ISIS Neutron and Muon Source, Rutherford Appleton Laboratory, Didcot OX11 0QX, United Kingdom; § Procter & Gamble, Brussels Innovation Centre, Temselaan 100, 1853 Strombeek-Bever, Belgium; ∥ Procter & Gamble, Newcastle Innovation Centre, Newcastle upon Tyne NE12 9TS, United Kingdom

## Abstract

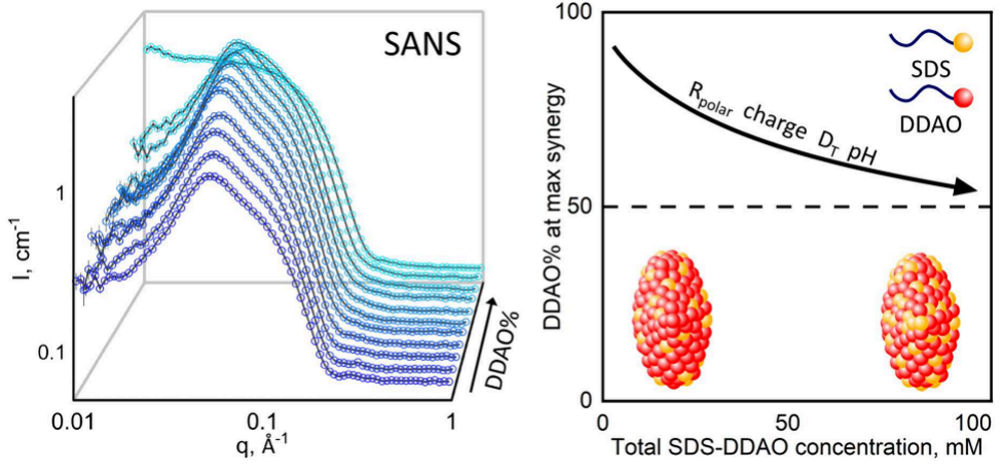

We investigate the structure and interactions of a model anionic/amphoteric mixed surfactant micellar system, namely, sodium dodecyl sulfate (SDS) and *N*,*N*-dimethyldodecylamine *N*-oxide (DDAO), employing SANS, FTIR, DLS, and pH measurements, in the range 0.1–100 mM total surfactant concentration and 0–100% DDAO. Increasing surfactant concentration is found to elongate the prolate ellipsoid micelles (*R*_Polar_ ∼ 25–40 Å), accompanied by up to a 6-fold increase in micellar charge. The surfactant synergy, in terms of micellar charge and size, diffusion coefficient, solution pH, and headgroup interactions, was found to vary with concentration. At lower concentrations (≤50 mM), the SDS–DDAO ratio of maximum synergy is found to be asymmetric (at 65–85% DDAO), which is rationalized using regular solution theory, suggesting an equilibrium between Na^+^ dissociation, DDAO protonation, and counterion concentration. At higher concentrations, maximum synergy shifts toward the equimolar ratio. Overall, our study expands and unifies previous reports, providing a comprehensive understanding for this model, synergetic mixed micellar system.

## Introduction

Mixtures of surfactant solutions are ubiquitous in everyday practical applications and industrial processes,^1^ harnessing surfactant synergies that enhance a range of properties with respect to those of the pure components (e.g., depressing the critical micelle concentration (CMC) and interfacial tension and enhancing detergency).^1−3^ Synergy is generally rationalized in terms of the formation of mixed surfactant layers at interfaces and in mixed micelles,^4−7^ governed by thermodynamics and architecture of the surfactant molecules (headgroups, tails, and branching), counterions, and solution medium.^5,6,8^ In the micellar phase, synergistic surfactants generally lead to increased aggregation numbers, micelle sizes, and charge/interactions^9−12^ as well as altering the concentration at which precipitation^13,14^ and spherical-to-worm-like micelle transitions^15,16^ have been observed. An understanding of these mechanisms and the structure and interactions of solution assemblies is crucial in surfactant formulation design.^2^

**Figure 1:**
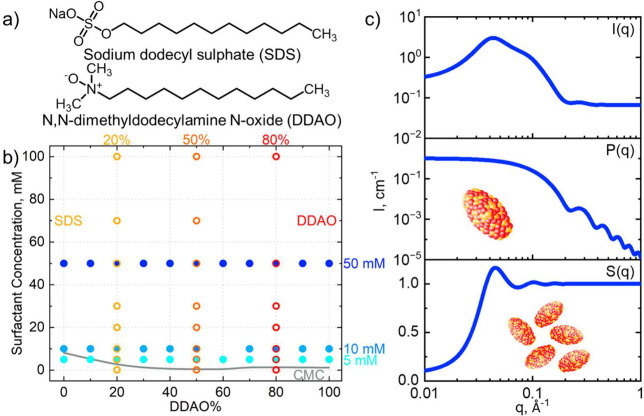
(a) Structures of SDS and DDAO surfactants. (b) Experimental concentration space investigated in SANS for SDS–DDAO mixtures in heavy water (D_2_O). The solid gray line indicates CMC values reported by Tyagi et al.^6^ (c) Illustration of SANS scattering profile *I*(*q*) (top), associated prolate ellipsoid form factor *P*(*q*) (middle), and Hayter–MSA structure factor *S*(*q*) (bottom), computed in SASView, characterizing the shape, size, and interactions of mixed micelles.

In this study, we investigate the structure, interactions, and mechanism for synergy in sodium dodecyl sulfate (SDS) and *N*,*N*-dimethyldodecylamine *N*-oxide (DDAO) mixtures in aqueous solution (Figure 1a). The pure components have been extensively studied^17−20^ as well as the phase boundaries for the mixed system (including precipitation and crystallization) and the role of pH,^13,21,22^ DDAO protonation upon SDS addition,^6,15,21,23^ and solution viscosity.^16^ Comparatively little has been reported about the structure and interactions of the mixed micelle system. Employing neutron scattering, Khodaparast et al.^24^ performed a study on 20% w/w (∼0.7 M) SDS solutions doped with up to 5% w/w DDAO at varying temperature, and Kakitani et al.^11^ investigated 80 mM total surfactant concentration solutions from 0 to 100% DDAO. Previous work largely suggests a “symmetric” synergy for this system, where CMC, surface tension, and micelle structure and interactions (at higher concentrations) exhibit maximum deviation from the pure components at the equimolar (50:50) mixture.

Here, in exploring the structure and interactions of micelles in the 0.1–100 mM range and 0–100% DDAO (encompassing the CMCs of DDAO, 1.1 mM, and SDS, 8.2 mM^6^), depicted in Figure 1b, we demonstrate that the synergy in micelles becomes asymmetric with respect to the DDAO content in decreasing the total surfactant concentration. Small-angle neutron scattering (SANS) measurements were performed for (i) constant surfactant ratio isopleths from 0.1 to 100 mM at fixed 20, 50, and 80 mol % DDAO ratios and (ii) constant total surfactant concentration isopleths from 0 to 100 mol % DDAO for 5, 10, and 50 mM concentrations. Dynamic light scattering (DLS) and pH measurements were performed to examine micellar diffusion and interactions in solution. Regular solution theory was then used to interpret the experimental data and suggest an underpinning mechanism to understand the asymmetric synergy based on DDAO protonation and surfactant partitioning in mixed micelles at varying ratios. Finally, Fourier transform infrared spectroscopy (FTIR) is employed to resolve molecular interactions and corroborate our findings before the SDS:DDAO ratio where the maximum synergy measured is discussed as a function of concentration, outlining this previously unreported phenomenon, important in the formulation engineering of surfactant solutions.

## Experimental Section

### Materials

Sodium dodecyl sulfate (SDS, BioReagent, ≥98.5%, Sigma-Aldrich, 151213) and *N*,*N*-dimethyldodecylamine *N*-oxide (DDAO, BioXtra, ≥99.0%, Sigma-Aldrich, 1643205) were used as received. For SANS measurements, stock solutions of the pure surfactants were prepared gravimetrically in heavy water (D_2_O, filtered, 99.8 atom %, Sigma-Aldrich, 7789200) and mixed on a tube rotator for 24 h. These were then mixed volumetrically to make the samples detailed in Figure 1b and similarly left to mix for 24 h prior to measurements. Samples for FTIR and DLS measurements were made following a similar procedure, however using *N*,*N*-dimethyldodecylamine *N*-oxide (DDAO, 30 wt % in H_2_O, Sigma-Aldrich, 1643205) as received and ultrapure 18.2 kΩ cm water filtered through a 0.2 μm cellulose acetate membrane (Sartorius), and were left to mix on a roller mixer.

### Small-Angle Neutron Scattering (SANS)

SANS measurements were performed at the ISIS pulsed neutron and muon source (Oxfordshire, UK) using the time-of-flight SANS2D diffractometer, with an incident wavelength range of 1.75–16.5 Å at 10 Hz and two detectors at a distance 2.4 and 4 m from the sample, yielding an approximate wavenumber, *q*, range *q* = (4π/λ) sin(θ/2) of 0.005–1 Å^–1^, where λ is the neutron wavelength and θ is the scattering angle.

Samples detailed in Figure 1b were transferred into 2 mm (for surfactant concentrations >10 mM) or 5 mm (≤10 mM) path length quartz glass banjo cells (Hellma 120-QS) and loaded into a temperature-controlled sample changer before SANS (simultaneous scattering and transmission) acquisition at 25 °C using a 12 mm circular aperture. MANTID software^25^ (v6.8.0) was used to bin, merge, radially average, and reduce the scattering data, scaled to absolute units (cm^–1^) using an isotopic polystyrene blend of known radius of gyration,^26^ as a secondary standard. SANS data *I*(*q*) were then analyzed with SASView (v5.0.6)^27^ using an ellipsoid model^28^ for the form factor, *P*(*q*), and a Hayter–MSA model^29,30^ for the interactions of micelles as the structure factor, *S*(*q*), illustrated in Figure 1c.

### Dynamic Light Scattering (DLS)

DLS measurements were performed using the VASCO KIN (Cordouan Technologies) system employing the in-situ head attachment with a laser diode (λ = 638 nm) and fixed 170° scattering angle (*q* ≈ 0.0026 Å^–1^). Measurements were directly taken from 1, 5, 10, and 50 mM samples of varying surfactant ratios in glass vials (28.25 mL, SAMCO T101/V7), with >10 repeats per sample, the temperature monitored by a Pt100 sensor, and the path length manually adjusted to ≈5 ± 1 mm inside the vial. Correlograms were fitted and analyzed using the integrated Sparse Bayesian learning (SLB) algorithm^31^ suitable for polymodal and continuous particle populations, yielding a decay constant, Γ = *D*_T_*q*^2^, from which the translational diffusion coefficient, *D*_T_, was calculated. The solution pH was also measured.

### Fourier Transform Infrared Spectroscopy

Attenuated total reflectance Fourier transform infrared (ATR-FTIR) spectroscopy measurements were performed using a Bruker INVENIO-S system and a DTGS detector. Spectra were averaged over 64 single beam scans with a 4 cm^–1^ resolution in the range 4000–1000 cm^–1^. Surfactant samples at 50 and 100 mM and varying component ratios were measured by transferring a 0.5 mL aliquot onto a single reflection diamond crystal (Platinum ATR accessory). The diamond crystal was cleaned and dried between measurements, for background acquisition, and in obtaining the solvent (H_2_O) spectrum. Results were processed in absorbance, using the OPUS 8.5 software with spectra being baseline corrected, normalized, solvent subtracted, and smoothed (due to the low concentrations used) with no further processing.

## Results and Discussion

### Pure Components

**Figure 2:**
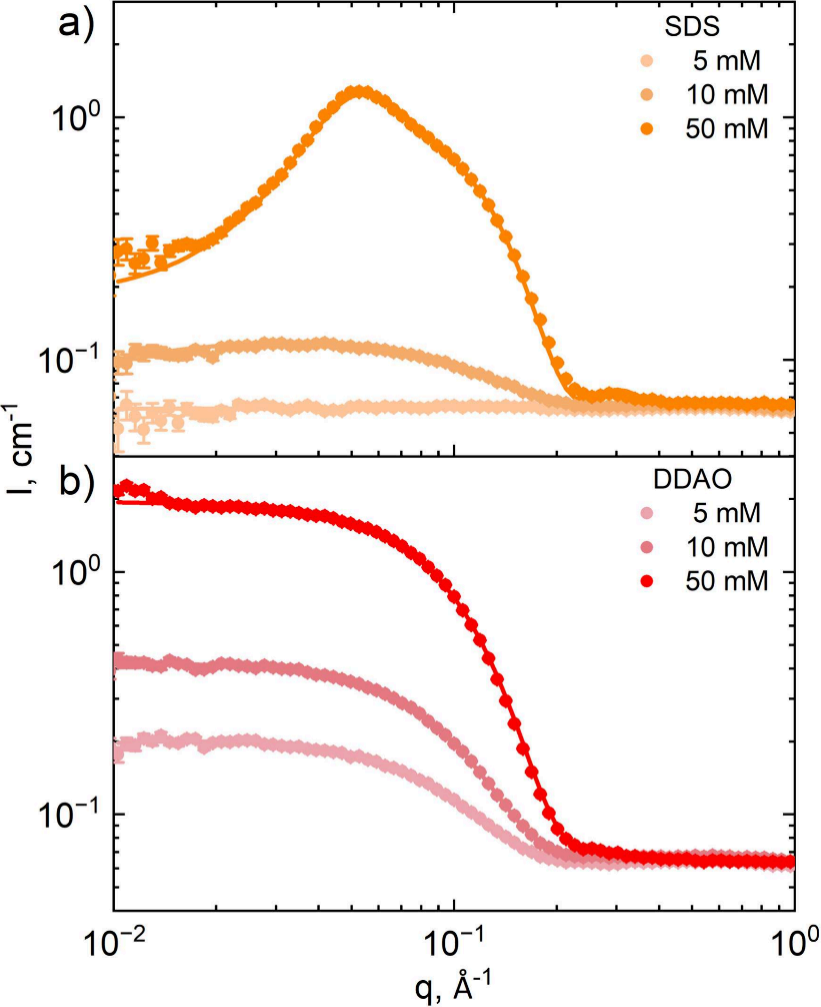
SANS scattering profiles for the pure components (a) SDS (anionic) and (b) DDAO (zwitterionic) in D_2_O at 5, 10, and 50 mM. Solid lines correspond to fits using ellipsoid form factors and Hayter–MSA structure factors (fitting parameters compiled below). The flat profile at 5 mM SDS is <CMC (≃8.2 mM for SDS). The CMC for DDAO is ≃1.1 mM,^6^ yet micelles have zero overall charge in neat aqueous solution.

Figure 2 shows the scattering data and fits obtained for pure SDS (a) and pure DDAO (b) solutions. At 5 mM, SDS and DDAO solutions are below and above the CMC, as seen by the absence and presence, respectively, of the micellar ellipsoid form factor. The SDS data show an emerging structure factor upon increasing concentration, as expected by an anionic surfactant; by contrast, DDAO is zwitterionic,^21,32^ with zero overall charge, and no structure factor contribution is present, even at 50 mM, well above CMC ≃ 1.1 mM.^6^ The SDS scattering profiles show the expected micelle formation at 10 mM (above the CMC of SDS, 8.2 mM)^6,21,33,34^ with the presence of a form factor and further presence of a significant structure factor at 50 mM due to the dissociation of Na^+^ in solution, generating negatively charged micelles and leading to an intermicelle electric double-layer interaction.^35−37^ Details of physicochemical parameters are given and compared to mixtures in the following section.

Self-consistency checks of our fitting procedure and fitting parameters are included in the Supporting Information (Figures S1 and S2). In this work, we fixed the SLD for the surfactant systems to that of the tail groups. This gave the most self-consistent results and minimized scatter in other fitting parameters at these low concentrations. We also note that our ellipsoidal form factor model is in contrast to previous studies that used a core–shell ellipsoid form factor for this system,^11,13,24^ albeit at higher concentrations. However, due to a greater scatter in other fitting parameters obtained from trialling the core–shell model and uncertainty on the SLD of the “shells” (headgroups), as will become evident when regular solution theory results are discussed, we therefore report the SANS data fitted with an ellipsoid form factor and an SLD of −0.691 × 10^–6^ Å^–2^, corresponding to the surfactant tails. SANS data fits yield an average χ^2^ ∼ 20, with χ_max_^2^∼ 100 at 20% DDAO and 100 mM total surfactant concentration.

### Isopleths of Constant SDS:DDAO Ratio

**Figure 3:**
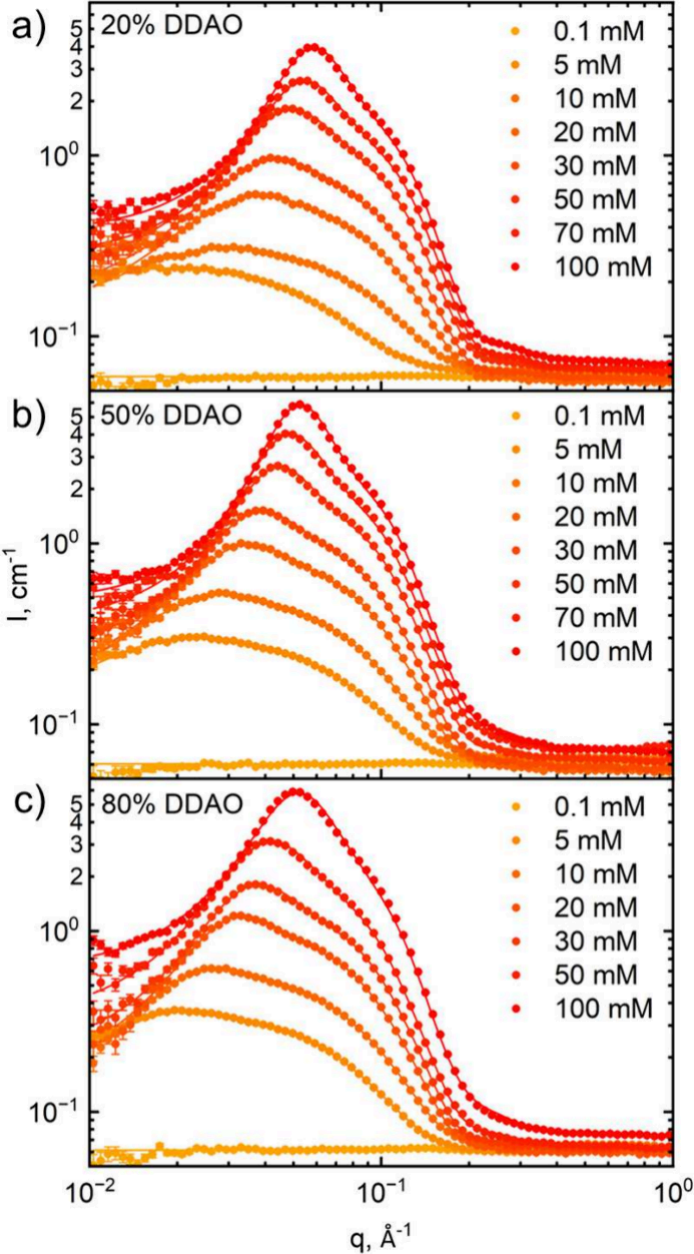
SANS scattering profiles for fixed SDS:DDAO ratios (a) 80:20, (b) 50:50, and (c) 20:80 in D_2_O over total surfactant concentrations 0.1–100 mM. Solid lines are fits to ellipsoid form factors and Hayter–MSA structure factors, and flat lines (0.1 mM) correspond to solutions below the CMC, included as a reference.

**Figure 4:**
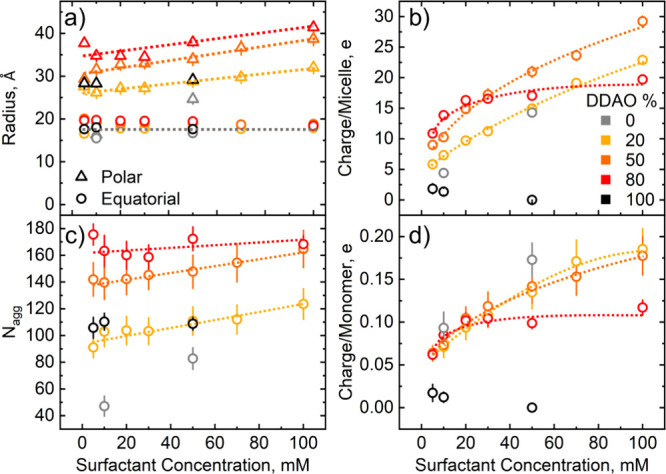
SANS fitting parameters for fixed ratio isopleths SDS:DDAO (100:0, 80:20, 50:50, 20:80, and 0:100) as a function of surfactant concentration (0–100 mM): (a) micellar radii (△, polar; ○, equatorial), (b) micellar charge inferred from Hayter–MSA structure factor, (c) aggregation *N*_agg_, and (d) effective charge per monomer (≡ charge/*N*_agg_). Error bars in (a, b), obtained from SASView fits, are smaller than the data points; uncertainties in (c, d) are calculated by error propagation.

Scattering profiles and fits for the constant surfactant ratio isopleths investigated are shown in Figure 3 for (a) 20%, (b) 50%, and (c) 80% DDAO over a 0.1–100 mM concentration range. For all ratios, a flat scattering profile can be observed at 0.1 mM (<mixed CMC),^6^ which arises from the, largely, incoherent scattering from monomers and a significant contribution from the solvent. Micellar solution profiles were fitted with an ellipsoid form factor and Hayter–MSA structure factor to extract parameters related to micelle size, shape, and interactions, as shown in Figure 4.

Figure 4a shows the polar (triangles) and equatorial (circles) radii for both the pure surfactants and constant ratio isopleth solutions. In line with previous work,^13,24^ we find that SDS–DDAO micelles are best described by prolate ellipsoids (*R*_equatorial_ < *R*_polar_). These data show that the prolate ellipsoid micelles elongate with increasing concentration, where the equatorial radius remains approximately constant, while the polar radius increases. This same trend is not seen for pure DDAO micelles in the 5–50 mM concentration range explored; however, the results show a general increase in equatorial radius when SDS is mixed with DDAO up to a maximum at 80 mol % DDAO.

Similarly, Figure 4b shows an increase in micelle interactions (interpreted as micelle charge through the Hayter–MSA structure factor model) with the concentration. Here, by virtue of an increase in micellar charge, the mixing of SDS and DDAO micelles augments micellar interactions when compared to the pure components. Furthermore, these data suggest a “crossover” region at around 30 mM where the mixed micelle ratio of highest charge switches from the 80% to the 50% DDAO solutions as the 80% DDAO micelles plateau in charge with concentration.

Aggregation numbers were calculated by dividing the volume of the corresponding prolate ellipsoid by the molecular volume of dodecane, *V*_tail_, calculated using the Tanford equation^38^*V*_tail_ = 24.7 + 26.9*n*_c_, where *n*_c_ is the number of carbons. Here, the headgroups were not considered due to the use of the surfactant tail SLDs for the fits. The change in aggregation numbers with concentration is shown in Figure 4c with, unsurprisingly, the same trends as those of the radii data. These were then used to calculate an effective charge per monomer (effective surface charge density) as shown in Figure 4d. Here, we see a decrease in the charge per monomer when DDAO is added to SDS. This is interpreted as due to a decrease in the density of SDS sulfate headgroups on the surface of the micelle by virtue of DDAO being integrated, carrying either a neutral or positive charge. These data also show that the surface charge density is similar for both 20% and 50% DDAO solutions with the 80% solutions plateauing at around 20 mM, being nearly 50% lower than the other ratios at 100 mM.

The reported micelle size, charge, and aggregation numbers are in good agreement with previous findings for pure SDS^8,12,17,18,20^ and DDAO^19,20,39^ solutions also studied in D_2_O. The use of D_2_O, instead of H_2_O, has been reported to result in a small increase of aggregation number of charged micelles, while preserving surface charge density (thus increasing overall micelle charge) and a slightly lower CMC.^40,41^ Overall data trends were found to be unchanged in both light and heavy water.

From this work, it can be seen that the evolutions of size, charge, aggregation number, and charge density follow similar trends to that of the pure SDS, however, with asymmetric changes in crossing between isopleths, i.e., maximum equatorial radius and micellar charge at 80% DDAO over the whole concentration range and <30 mM, respectively. This asymmetry is more evident and refined along constant concentration isopleths.

### Isopleths of Constant Total Surfactant Concentration

**Figure 5:**
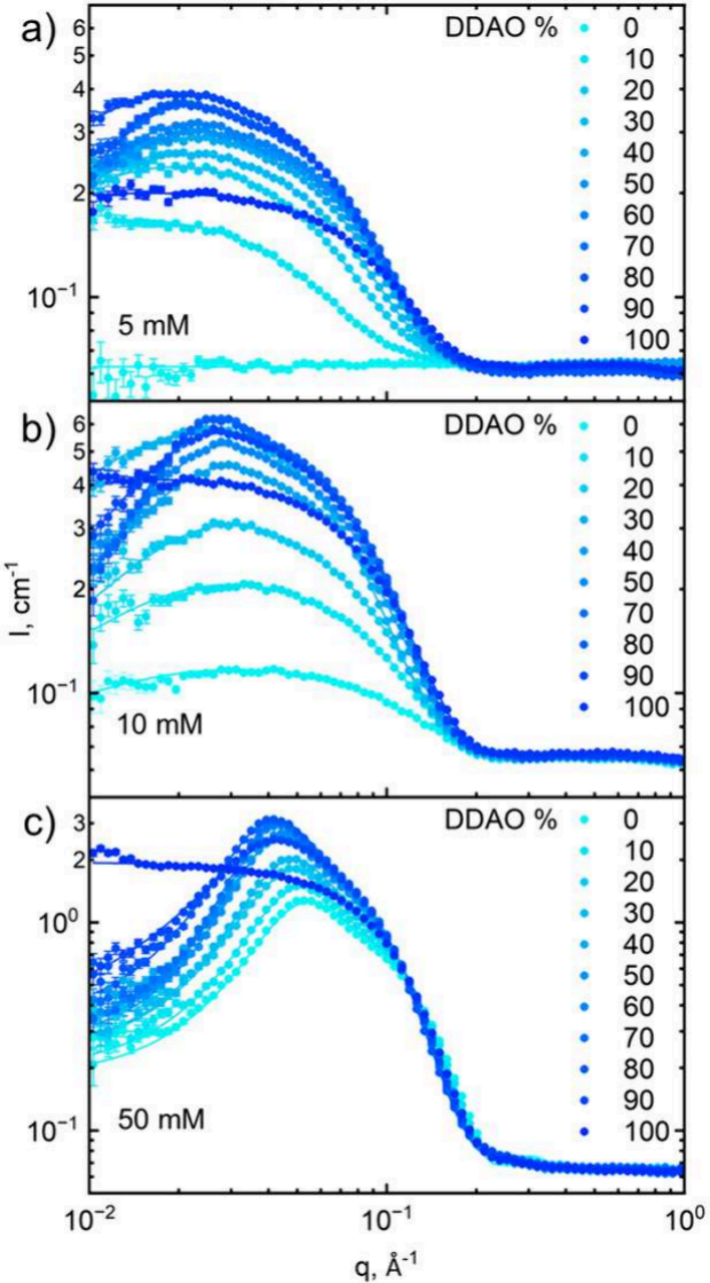
SANS scattering profiles for mixed SDS:DDAO concentrations (a) 5, (b) 10, and (c) 50 mM in D_2_O, at varying surfactant molar ratios from 0 to 100% DDAO. Solid lines are fits to ellipsoid form factors and Hayter–MSA structure factor. The flat profile at 5 mM and 0% DDAO (neat SDS) is <CMC (≃8.2 mM for SDS^6^).

**Figure 6:**
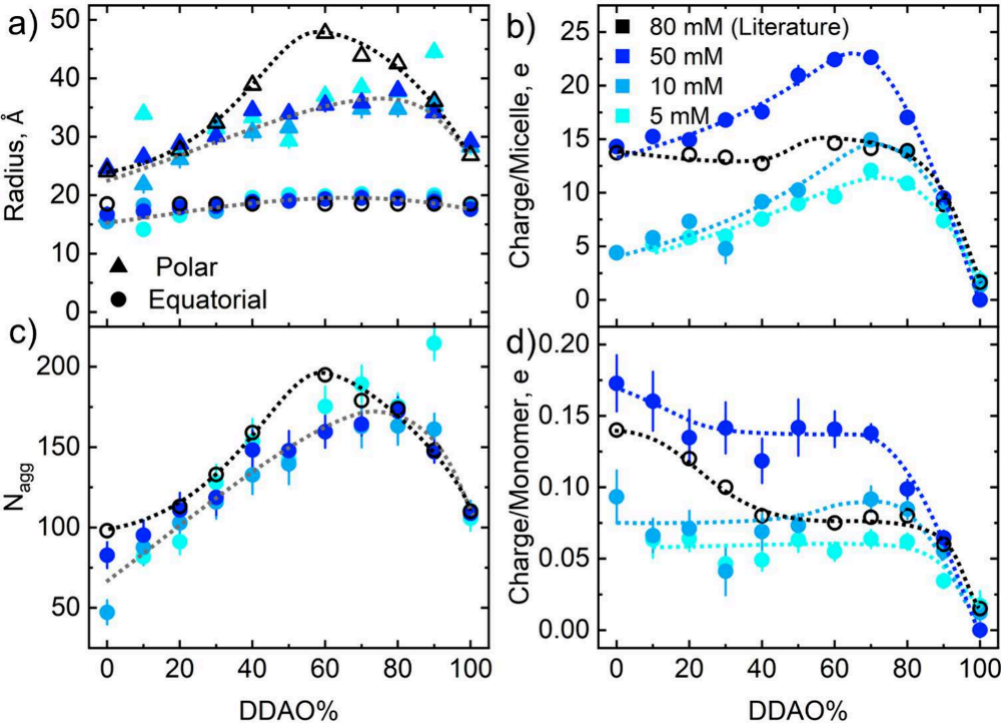
SANS fitting parameters for fixed concentration isopleths (5, 10, and 50 mM) and varying SDS:DDAO molar ratios: (a) micellar radii (△, polar; ○, equatorial), (b) micellar charge inferred from the Hayter–MSA structure factor, (c) aggregation *N*_agg_, and (d) effective charge per monomer (≡ charge/*N*_agg_). Data points at 80 mM replotted from Kakitani et al.^11^ Error bars in (a, b), obtained from SASView fits, are smaller than the data points; uncertainties in (c, d) are calculated by error propagation.

Figure 5 shows the scattering profiles of constant total surfactant concentration isopleths at (a) 5 mM, (b) 10 mM, and (c) 50 mM in the range 0–100 mol % DDAO investigated. The reduction in CMC of mixed surfactant systems, previously reported,^6^ is apparent from the scattering profiles measured at 5 mM total surfactant concentration, with a profile indicative of ellipsoidal micelles appearing upon dosing as little as 10 mol % DDAO. For all concentrations, the profiles show an increase in the scattering intensity with increasing DDAO% up to ∼70–90% DDAO and decreasing toward pure DDAO thereafter. The solid lines represent fits to an ellipsoid form factor, Hayter–MSA structure factor, imposing a prolate ellipsoid geometry and utilizing the SLDs of the surfactant tails, with resulting fitting parameters and those from Kakitani et al.^11^ shown in Figure 6.

Figure 6a shows the polar (triangles) and equatorial (circles) radii of the fitted ellipsoid micelles at 5, 10, 50, and 80 mM over the range of 0–100 mol % DDAO. At 5–50 mM, there is little change in radii between concentrations; hence, the guides to the eye show the general trend of these data. These data show a maximum in the polar radius of micelles in the range 75–85% DDAO, decreasing with increasing concentration, and a nearly constant (albeit with a slight maximum) equatorial radius, implying an asymmetric elongation of micelles up to these skewed maxima away from the equimolar composition found by Kakitani et al. at 80 mM as shown.^11^ This asymmetry is also observed in the micellar interactions through the charge per micelle data shown in Figure 6b. Here, the maximum charge/interaction is observed at the tighter range of 65–70% DDAO for 5, 10, and 50 mM concentrations, with 80 mM data showing a slight maximum near the 50:50 mixture. These data also suggest that upon increasing concentration, the addition of DDAO ceases to lead to an increase in micellar interactions and instead leads to a slight decrease, in contrast to what is observed at the lower concentrations from 0% DDAO to the maxima.

Figure 6c shows the aggregation numbers of micelles calculated from the micellar radii as previously explained, where the same asymmetric maximum is observed. In calculating the charge per monomer (effective surface charge density), it can be seen from Figure 6d that these maxima in size and charge are instead converted to a “turning point” where for 5 and 10 mM solutions the surface charge density remains approximately constant between 0% and ∼80% DDAO (with a small increase for the 10 mM isopleth) before rapidly dropping to ∼0 *e* at 100% DDAO. For the 50 and 80 mM data, this rapid decrease is also observed at ∼70–80% DDAO; however, there is now what could be interpreted as a small decrease in surface charge density between ∼0 and 40% DDAO.

These findings are in good agreement with the work of Khodaparast et al.^24^ whose data showed an elongation in the prolate micelles, of 20% w/w (∼0.7 M) SDS solutions with increasing DDAO concentration up to 5% w/w. Furthermore, over the same concentration range, it was reported that the monomer charge decreased with DDAO addition, also observed in this work for the 50 mM isopleth between 0 and 30%, suggesting a change in the trend of monomer charge density (or surface charge density) with DDAO% between 10 and 50 mM. For two cationic surfactants, Lusvardi et al.^10^ observed an increase in micelle radius and aggregation number with increasing DTAB% (up to 30%) in a DTAB–DDAB system. However, for SDS and DBNMG, a SANS and fluorescence study by Griffiths and co-workers^9^ showed that the micellar charge decreases with added nonionic surfactant. This synergistic increase in micelle size and interactions is therefore not specific to the SDS–DDAO system and is also observed in surfactants with the same charge; however, it does not necessarily hold for all ionic–nonionic surfactant systems.

**Figure 7:**
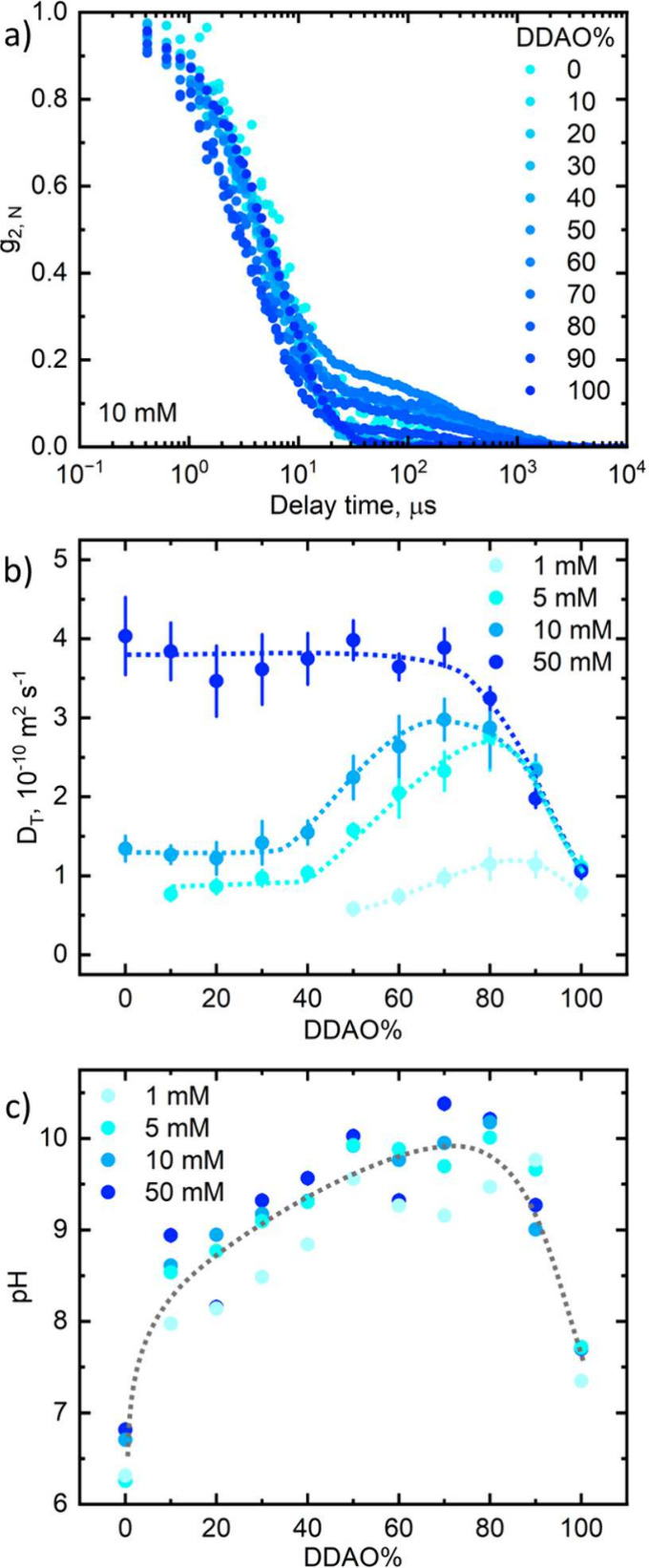
(a) Example normalized intensity autocorrelation functions (*g*_2,*N*_ ≡ (*g*_2_ – *B*)/(*B*β)) obtained by DLS for 10 mM SDS:DDAO surfactant solutions of varying molar ratios (0–100%). (b) Apparent translational diffusion coefficients (*D*_T_) for 1–50 mM concentration and varying SDS:DDAO molar ratios, inferred from SBL fitting of the fast decay of the DLS correlograms, Γ = *Dq*^2^, where Γ is the decay constant. (c) pH of the SDS–DDAO solutions measured in DLS for 1–50 mM concentrations and 0–100% DDAO composition. Error bars in (b, c) were calculated from 5 repeats; the maximum uncertainty in pH measurements was ±0.05.

In terms of the asymmetric synergy observed at lower concentrations, Kakitani et al.^11^ concluded that from a SANS study, for this SDS and DDAO system at 80 mM, the size, charge, and aggregation numbers of micelles exhibit a maxima at the equimolar mixture. This symmetric maximum synergy composition is also observed in other mixed surfactant micelle systems. Prevost et al.^12^ reported that the aggregation number of a 200 mM SDS and DTAC solution increased toward the 50/50 mixture before precipitation. This and the findings in this work suggest that this asymmetric synergy is both system- and concentration-dependent. To further investigate this, DLS measurements were performed on 1–50 mM SDS–DDAO solutions of varying ratios with results shown in Figure 7 along with pH data taken immediately after DLS measurements to give insights into the protonation state of DDAO.

Figure 7a shows normalized intensity autocorrelation functions for the 10 mM systems at ratios between 0 and 100% DDAO. An SLB fitting analysis was performed to extract the “apparent” translational diffusion coefficient, *D*_T_, of surfactant micelles from the first decay, as shown in Figure 7b. Data for 1–10 mM show an increase in apparent diffusion coefficient with DDAO addition up to a maximum at 70–90% with the peak shifting toward a lower DDAO content with increasing concentration. For the 50 mM isopleth, the diffusion coefficient remains approximately constant within the error bars, before decreasing at around 60–70% DDAO. These data show, perhaps, more clearly how the maximum for this physicochemical property shifts toward the equimolar ratio with increasing concentration. This increase in apparent translational diffusion coefficient has been observed and explained previously as DLS measures the mutual diffusion coefficient of micelles, monomers, and counterions in solution.^42−45^ For uncharged micelles in solution, such as those formed by DDAO in neutral to basic conditions, the mutual diffusion coefficient equals the micellar diffusion as can be seen at all concentrations investigated from Figure 7b at 100% DDAO, where the near constant micelle radii from SANS for DDAO micelles with concentration are supported by little to no change in diffusion coefficient. However, for SDS micelles, which increase in polar radius from 16 Å at 10 mM to 25 Å at 50 mM, we observed an increase in apparent diffusion coefficient. For the case of surfactants that become charged in solution, the mutual diffusion coefficient is now a weighted average of charged monomers, counterions, and micelles.^43^ Therefore, Figure 7b displays an increase in micellar charge, counterion dissociation (Na^+^ from the SDS headgroups), charged monomers in solution, and a stronger interaction between them. This supports our SANS findings of an asymmetry in the SDS–DDAO micellar interactions and charge through a change in the contributions to the mutual diffusion coefficient measured by DLS.

Figure 7c shows the pH of the 1–50 mM SDS–DDAO solutions over a 0–100% DDAO range used for DLS measurements. We note that pD values of D_2_O solutions are generally slightly higher that in H_2_O, but only by a few percent.^46,47^ These results also show an asymmetric increase in pH due to the mixing of the two surfactant solutions into mixed micelles with the highest pH measured at around 70–80% DDAO—the region of maximum interaction. This increase in pH toward alkaline conditions has been attributed to the synergistic effect of both the sulfate and amine oxide headgroups, whereby the anionic sulfate group increases the concentration of hydronium, H_3_O^+^, counterions in the diffuse layer of the micelle, leading to the protonation of the amine oxide and reduction of H_3_O^+^ in solution^6,15,23^ albeit a previously measured p*K*_a_ ≈ 5 for the amine oxide headgroup of pure DDAO solutions.^21^ This would suggest a decrease in micelle charge and interactions with increasing pH, as the resulting cation should screen the anionic charge on the sulfate group. To rationalize the effect on the solution pH of mixing surfactants and formation of mixed micelles with the observed parameters form SANS fits and DLS measurements, we reinterpret our data through the lens of regular solution theory.

### Regular Solution Theory

The treatment of mixed surfactant systems as regular solutions where components may have synergistic or antagonistic interactions was initially developed by Rubingh et al.^48,49^ Through this, information on micellar composition, mixed CMC, and the interaction parameter, β, may be obtained with knowledge of the other parameters.^50−52^ In this work, we are concerned with micelle composition, which may be estimated from
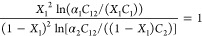
1where *X*_1_ and α_1_ are the mole fractions of surfactant 1 in a binary mixed micelle and total mixed surfactant (solute), respectively, and *C*_*i*_ and *C*_12_ are the CMCs of the pure components *i* and the mixed surfactant system, respectively. With knowledge of the CMCs of mixed and pure components and the solute mole fraction of one of the components, eq 1 may be solved iteratively to predict the mole fraction of both surfactants in mixed micelles.

**Figure 8:**
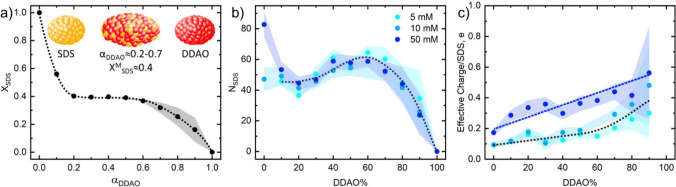
(a) Calculated average SDS mole fraction in mixed micelles (*X*_SDS_^M^) as a function of total (solute) DDAO mole fraction (α_DDAO_) from regular solution theory (eq 1) utilizing pure and mixed CMC values previously reported by Tyagi et al.^6^ (b) Average number of SDS monomers per mixed micelle (*N*_SDS_^M^ ≡ *N*_agg_*X*_SDS_^M^) in solutions of 5, 10, and 50 mM total surfactant concentration and varying, 0–100%, DDAO molar ratios. (c) Mean effective charge per SDS monomer in a mixed micelle (≡ charge/*N*_SDS_^M^) in solutions of 5, 10, and 50 mM total surfactant concentration. Shaded regions represent the error in extrapolating mixed CMC values^6^ in (a–c) combined with the propagated error from aggregation numbers and charge in this work in (b, c).

In this work, CMC values reported by Tyagi et al.^6^ were fit using different polynomials to predict the SDS mole fraction in mixed micelles, *X*_SDS_, as a function of the mole fraction of total DDAO solute, α_DDAO_, shown in Figure 8a, with shaded regions showing the range of values obtained from the fits to the CMCs. These calculations predict that the composition of mixed micelles is near constant at ∼0.4 SDS mole fraction in the range of ∼0.2–0.7 DDAO solute mole fraction. This corresponds to a region where SANS results in Figure 6 predict (a) an elongation of the prolate ellipsoid micelles, (b) an increase in micelle interactions, interpreted as micelle charge, (c) an increase in aggregation number, and (d) an approximately constant surface charge density.

To further examine these calculations, the micellar aggregation numbers reported here were used to calculate the aggregation number of SDS molecules in the mixed micelles, *N*_SDS_, shown in Figure 8b. These results show that although the amount of SDS solute dissolved in solution decreases, the increase in aggregation number of micelles as a function of DDAO content leads to an overall increase in the number of SDS molecules present in the elongating micelles up to the maximum at around 60–70% DDAO. Furthermore, these numbers were used to calculate an effective charge per SDS molecule in the mixed micelle, shown in Figure 8c.

This effective charge is a function of Na^+^ dissociation from the sulfate headgroup, the counterion concentration in the electric double layer of the micelles, and the degree of DDAO protonation. With these results from regular solution theory and the conclusions from SANS fittings in Figure 6, we propose the following mechanism to account for the observed synergy: (i) As DDAO is added into the mixed micelle system, the total number of SDS molecules in the mixed micelle increases in the range 20–70% DDAO (Figure 8b). (ii) An increase in the number of sulfate headgroups, assuming constant Na^+^ dissociation, leads to more anionic headgroups per micelle and thus an increase in micellar charge. (iii) With the increased negative charge on the surface of micelles, the concentration of H_3_O^+^ at the micelle surface increases, leading to the protonation of DDAO,^6,15,23^ and an increase in the pH of the solution (Figure 7c), possibly aligning with our results of a constant surface charge density in the 20–70% DDAO range (Figure 6d) and overall increase in micelle interaction/charge due to an increase in SDS aggregation numbers (Figure 8b). (iv) However, calculations from regular solution theory suggest that the effective SDS charge increases with DDAO addition (Figure 8c); therefore, we propose that the presence of cationic DDAO headgroups now screens repulsions between anionic sulfate groups, shifting the equilibrium to a state where more Na^+^ dissociates. (v) In turn, this leads to an increase in negative surface charge, and therefore steps iii–iv would repeat until equilibrium is reached, accompanied by a pH increase due to DDAO protonation, thus lowering the H_3_O^+^ counterion concentration in solution and raising the concentration of OH^–^ which in turn associates with Na^+^ counterions, overall decreasing the screening of intermicelle interactions. (vi) The increase of average SDS charge by Na^+^ dissociation and reduced counterion availability reaches equilibrium with the degree of DDAO protonation which opposes this increase, thereby resulting in an effective overall constant surface charge density with DDAO addition (Figure 6d) and an overall increase in micellar interactions (Figure 6b) associated with the increase in SDS aggregation number and solution pH (Figure 7c) with maximum at 70 mol % DDAO.

This proposed mechanism strictly applies to the 5 and 10 mM concentration isopleths. Our findings for the 50 mM data and previous work at 0.7 M^24^ and 80 mM^11^ solutions show that the surface charge density appears to initially decrease with DDAO addition, and the DAAO% of maximum synergy moves toward the 50% solution. We tentatively interpret these observations as due to the high (>doubling) surface charge density of neat SDS at ∼50 mM, compared to 5–10 mM, for which up to 20% DDAO has a marginal impact on sulfate headgroup interactions, impeding step iv above. Moreover, upon increasing the concentration by 8 times to 80 mM, the return of the maxima in micellar properties to the equimolar ratio may be related to the total surfactant concentration with respect to the CMC of the pure components. The 5 and 10 mM solutions are respectively below and 1.2 times the CMC of pure SDS when compared to 10 times above for the 80 mM solution. The large excess of surfactants in the micellar phase at 80 mM may lead to a more expected direct correlation from solute to micellar partitioning.

To corroborate these hypotheses, we employ FTIR to examine the ordering of surfactant tails in the mixed micelles as a function of DDAO addition and assess whether the increase in SDS and DDAO charge can be observed through their headgroup interactions.

### FTIR and Molecular Interactions

**Figure 9:**
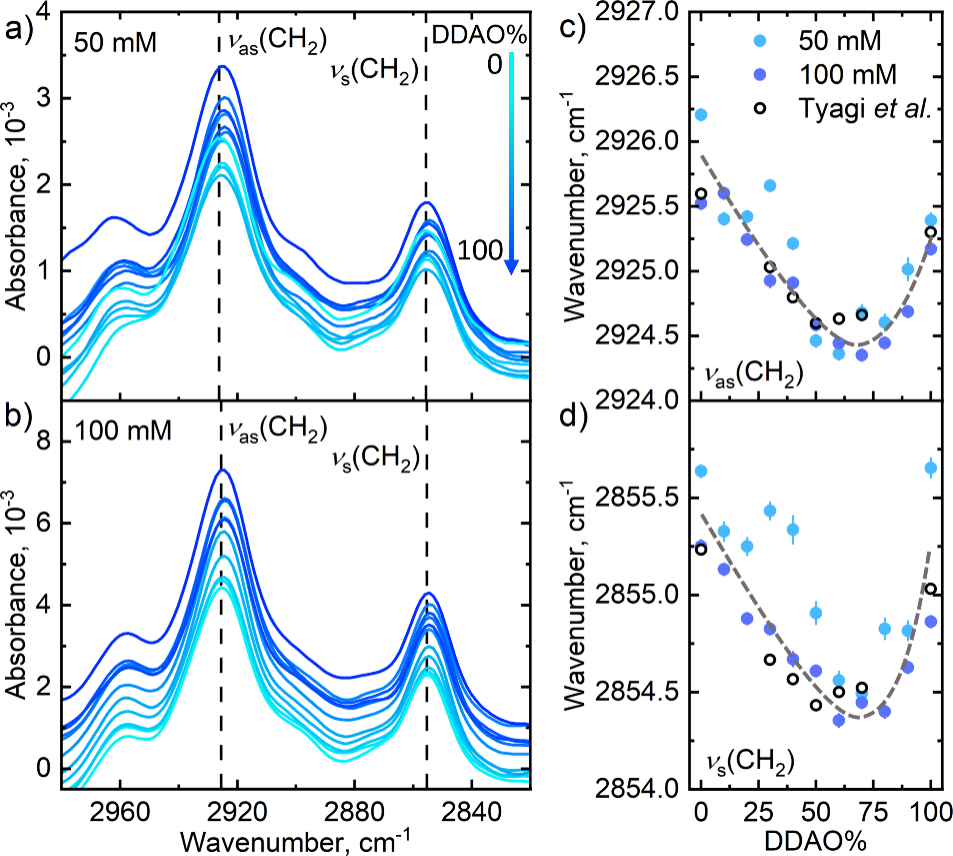
ATR-FTIR spectra of the antisymmetric and symmetric CH_2_ region for SDS:DDAO aqueous solutions of varying ratios (in steps of 10% DDAO) at (a) 50 mM and (b) 100 mM overall concentration. Dashed lines are located at the wavenumber of 0% DDAO (neat SDS) to highlight peak shifts. Shift in the wavenumber of the (c) antisymmetric and (d) symmetric stretches of 50 and 100 mM solutions over the range 0–100% DDAO (molar) including data replotted from Tyagi et al.^6^ at 100 mM.

FTIR spectra of SDS and DDAO mixed surfactant systems comprise two main regions of interest: absorption bands in the 3000–2800 cm^–1^ region due to vibrations of the methylene, CH_2_, groups of the surfactant tails and bands in the 1300–1100 cm^–1^ region, corresponding to sulfate, SO_3_, vibrations. Figure 9 shows the methylene absorption region for 50 mM (a) and 100 mM (b) total surfactant concentration, with the full spectra shown in Figure S3. Two main absorption bands are identified as the antisymmetric and symmetric CH_2_ vibrations at about 2926 and 2855 cm^–1^, respectively, with the dashed lines showing the peak positions for pure SDS solutions, highlighting their shift with DDAO addition. Such shifts have been associated with the crowding of surfactant tails in the micelle core, determined by their gauche/trans conformation ratio.^6,15,53^

The wavenumbers for these peaks and previous data at 100 mM by Tyagi et al.^6^ are shown in Figure 9 for the antisymmetric (c) and symmetric (d) absorption bands at 50 and 100 mM total concentration. These results show a shift in both vibration modes to lower frequency, associated with a conformational change of the surfactant tails from predominantly gauche, to an increased ratio in the trans state.^6^ This is interpreted as due to increased crowding and greater proximity and alignment of surfactant tails. Weers et al.^15^ noted that this frequency decrease follows the increase in aggregation number of the mixed micelles.

Given that the equatorial radius remains approximately constant with DDAO addition, we propose that this frequency shift is associated with the elongation of the ellipsoidal micelles. While surfactant molecules in regions of high curvature (i.e., near the “ends” of the ellipsoid) predominantly retain a gauche conformation, micelle elongation increases the area fraction of low surface curvature, which allows for chains to predominantly pack in a trans conformation. With increasing polar radius upon DDAO addition, the ratio of surfactants in trans compared to gauche conformation increases, leading to the decrease in frequency observed around the same 70% ratio.

**Figure 10:**
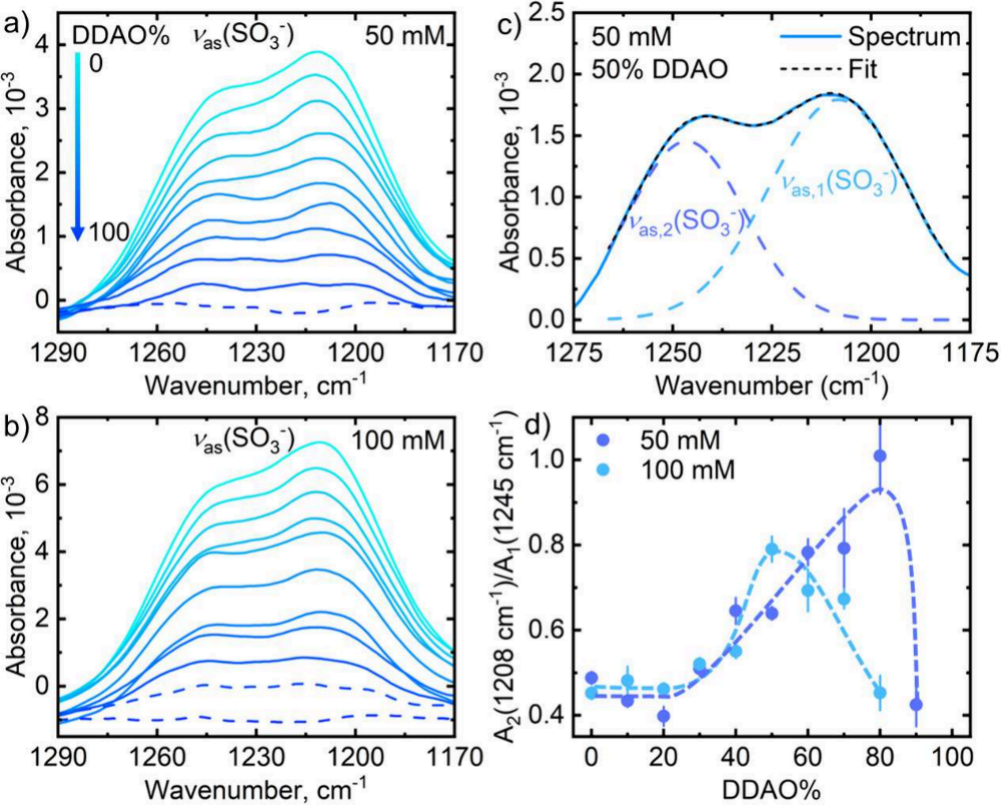
ATR-FTIR spectra of the antisymmetric sulfate region for SDS:DDAO aqueous solutions of varying ratios (in steps of 10% (M/M) DDAO) at (a) 50 mM and (b) 100 mM overall concentration in steps of 10% DDAO. (c) Example of a band deconvolution for 50:50 SDS:DDAO at 50 mM, employing Voigt profiles. (d) Change in the area ratio of band at 1208 cm^–1^ over area of the 1245 cm^–1^ band with DDAO composition.

Figure 10 shows the FTIR spectra of the antisymmetric sulfate absorption region for 50 mM (a) and 100 mM (b) total surfactant concentration with different spectra differing by 10% DDAO. In this region, there are two antisymmetric absorption peaks at around 1210 and 1240 cm^–1^ where for pure SDS solutions, it appears as a peak at 1210 cm^–1^ with a shoulder. Changes in the shape, intensity, and relative areas of these peaks provide information on the lateral interaction of the sulfate headgroup.^6,13,15,53^ This is because the transition dipole moment of the antisymmetric sulfate stretch is parallel to the micelle surface; hence, changes in these absorption bands lead to changes in headgroup interactions. To analyze sulfate headgroup interactions, a deconvolution was performed using a Voigt function as exemplified in Figure 10c for the 50 mM, 50% DDAO spectrum. The ratio of the lower:higher frequency was calculated and is plotted in Figure 10d showing changes in the relative contributions of the two antisymmetric stretches to the overall absorption region for 50 and 100 mM solutions. Changes in their relative contribution have been associated with changes in the symmetry environment around the sulfate headgroup whereby a strong interaction between SDS and DDAO headgroups leads to a further lowering in the symmetry of the sulfate group.^6,13^

Data for the 50 mM solution indicate the largest variations near 80% DDAO, corresponding to greater sulfate–amine oxide interaction. Our proposed cycle of Na^+^ dissociation and DDAO protonation (from regular solution theory) agrees with these FTIR findings as we expect a high proportion of both SDS and DDAO headgroups being charged. Furthermore, the increasing attractive interaction between surfactant heads leads to a decrease in area occupied by each headgroup and thus a closer packing of surfactant tails in the hydrophobic core, which would promote a trans conformation. We believe those data still more closely follow our reported findings on the polar radius of the micelle due to the differences in concentration maxima; however, it cannot be neglected that a stronger headgroup interaction would also affect the packing of the surfactant tails.

Figure 10d also shows data for 100 mM SDS–DDAO solution where a more symmetric relationship is observed, with greatest headgroup interactions near the equimolar composition. This change seemingly has no effect on the surfactant tail conformation (Figure 9c,d), further supporting a dependence on the degree of elongation of ellipsoidal micelles. Furthermore, this is in line with previous work on SDS–DDAO micellar system at higher concentration,^6,11,13^ which suggests a concentration dependence of headgroup interactions and therefore micellar charge, further corroborating a concentration dependence of the maximum synergy surfactant ratio.

**Figure 11:**
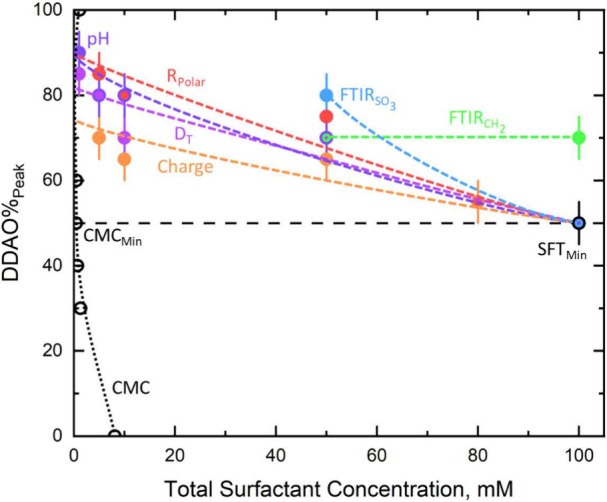
Concentration dependence of the SDS:DDAO ratio (in DDAO%_peak_) where the peak (maximum or minimum) is measured for the polar radius (red), micelle charge (orange), apparent translational diffusion coefficient (violet), pH (purple), CH_2_ absorbance wavenumber (green), and ratio of the areas of the SO_3_ antisymmetric stretches (blue). CMC and SFT data are from Tyagi et al.,^6^ and charge and polar radius data at 80 mM are from Kakitani et al.^11^

With all our findings outlined, we conclude by plotting the SDS:DDAO ratio (DDAO%_peak_) where the peak (maximum or minimum) in the physicochemical properties investigated is observed as a function of concentration, as shown in Figure 11. Figure 11 includes CMC and SFT data from Tyagi et al.^6^ and Kakitani et al.^11^ for the size and charge of micelles data at 80 mM. The plot shows the DDAO composition at which the maximum deviations from the pure components in the polar radius, micelle charge, apparent diffusion coefficient, pH, and FTIR analysis on the CH_2_ peak positions and SO_3_ peak area ratios were extracted from the model fits, measured, or analyzed. For each point, a 5% error bar is assigned associated with our measurement density (in steps of 10% DDAO), and the dashed lines are shown as a guide to the eye.

These data in Figure 11 show that the ratio of maximum synergy for the SDS–DDAO system tends toward the equimolar ratio from DDAO-rich compositions with increasing concentration. As discussed, our proposed mechanism (aided by regular solution theory) to explain our observations strictly applies to our data for low concentrations (5–10 mM) which is possibly due to how close the total surfactant concentration is to the CMC of SDS at 8.2 mM.^6^ We believe that the micellar composition calculated from regular solution theory would resemble more of a straight line on increasing concentration as the number of moles of pure SDS and DDAO micelles that are mixed become more comparable.

## Conclusions

We examine the structure and interactions of a model anionic/amphoteric mixed surfactant system, SDS–DDAO, employing a combination of scattering, spectroscopy, and thermodynamic modeling. SANS measurements reveal that the maxima in elongation, charge, and aggregration number of the prolate ellipsoid micelles occur in the range 70–90 mol % DDAO for 5–50 mM concentrations. A similar asymmetry is observed in DLS and pH measurements and is rationalized in terms of the surfactant partitioning in the mixed micelles, estimated from regular solution theory. We propose that the protonation of DDAO in SDS–DDAO mixed micelle systems^6,15,23^ leads to an increase in Na^+^ dissociation and reduction in counterion concentration to account for the micelle interactions measured and implied by SANS and DLS, respectively. FTIR measurements, when compared to observed trends extracted from SANS analysis, imply that the packing of surfactant tails in the mixed micelle is dependent on the degree of elongation, which, in turn, impacts the ratio of gauche/trans conformations of the surfactant tails. Examination of the absorption bands for the sulfate antisymmetric stretches reveals an increase in interaction between headgroups of the micelle, compatible with the mechanism of an increase in Na^+^ dissociation and DDAO protonation, and showing that this asymmetry decreases upon increasing concentration toward the equimolar ratio. Finally, we show that the composition (% DDAO) where maximum synergy is observed for the micellar physicochemical properties we measured is dependent on concentration, with a decrease in concentration toward the CMC leading to the maximum deviation from pure components tending toward the pure DDAO composition.

Our SANS data expand and complement the findings of Kakitani et al.^11^ where a higher concentration of 80 mM SDS–DDAO micellar system was found to exhibit near symmetric synergy with DDAO composition, suggestive of twin-tail (gemini) surfactant behavior between SDS and DDAO. Further, work by Prévost et al.^12^ and Tyagi et al.^13^ revealed that the precipitation boundary for higher concentrations of this system is symmetric and centered at the equimolar solution. Measurements of the kinematic viscosity of SDS–DDAO solutions by Safonova et al.^16^ corroborate a transition of the maximum viscosity from 80% to 50% DDAO with increasing total surfactant concentration in the 1–15% w/w range, and here we show that this transition also applies for a range of micellar structural and interaction properties.

Overall, we establish that the synergistic interactions in SDS–DDAO mixed micelles at low concentration are asymmetric with respect to the DDAO ratio and that this asymmetry decreases with increasing concentration away from the CMC. Therefore, in future works, investigations into the synergies present in surfactant systems and their potential application may need to consider this concentration dependence, especially in cases where micellar structure and interactions need to be controlled. Furthermore, these finding are relevant to the design of multicomponent surfactant systems, extensively employed in a range of formulations, from detergents^54,55^ to food^56^ and pharmaceutical products.^57^

## Supporting Information Available

Scattering length densities (SLD) employed in SANS fitting; surfactant volume fractions and scattering backgrounds; full FTIR spectra of surfactant solutions at 50 and 100 mM and 0–100% DDAO in 10% steps, highlighting tail and headgroup characteristic regions (PDF)
