# Supplementary material for: Concentration Dependent Asymmetric Synergy in SDS–DDAO Mixed Surfactant Micelles
Source: Langmuir. 2024 Mar 27;40(14):7433–43. doi: 10.1021/acs.langmuir.3c03900 (PMC11008254; doi:10.1021/acs.langmuir.3c03900)
Supplement: Supplementary file 1 — la3c03900_si_001.pdf [file la3c03900_si_001.pdf]

Supplementary Information

Concentration Dependent Asymmetric Synergy in SDS-DDAO Mixed Surfactant Micelles

Luis M. G. Torquato[a], Gunjan Tyagi[a], William N. Sharratt[a,b], Zain Ahmad[a], Najet Mahmoudi[c], Jérémie Gummel[d], Eric S. J. Robles[d], and João T. Cabral[a,*]

[a] Department of Chemical Engineering, Imperial College London, London, SW7 2AZ
[b] Current address: School of Engineering, University of Liverpool, Liverpool, L69 3GH, UK.
[c] ISIS Neutron and Muon Source, Rutherford Appleton Laboratory, Didcot, OX11 0QX, UK.
[d] Procter & Gamble, Brussels Innovation Centre, Temselaan 100, 1853 Strombeek-Bever, Belgium.
[e] Procter & Gamble, Newcastle Innovation Centre, Newcastle upon Tyne NE12 9TS, United Kingdom.
[*]j.cabral@imperial.ac.uk

1 SANS Data fitting parameters: SLDs

Our approach to fitting the SANS data was as follows. In the first stage, estimated ranges for the scattering length density, SLD, and volume fraction were calculated. The range of values for the SLD was estimated as ranging from that associated with the surfactant tails (minimum) and the surfactant tails, heads and Na^+ counterions (maximum); the volume fraction range was estimated between the total solute dissolved in D_2O (maximum) and minus the CMC using values from literature[1] (minimum), both converted into volume fraction assuming a density of 1.01 g cm^{-3}. This initial step revealed that the SLDs for both 5 mM and 10 mM data fluctuated non-linearly between the ranges given, while the 50 mM data consistently yielded an SLD representative of the surfactant tails in micelles. From this, we therefore decided to fix the micellar SLDs to -0.691 10^{-6} $Å^{-2}$, corresponding to the value of the surfactant tails (shared by DDAO and SDS), which fall within the uncertainty of all fitted SLD values. This procedure evidently reduces scatter in other fitting variables, and is discussed in the main paper.

2 SANS data fitting parameters: Volume Fraction

Figure S1 shows the volume fraction for (a) the fixed surfactant ratio and (b) fixed total surfactant concentration isopleths obtained from the data fitting on SASView. As expected, we find a linear increase in micellar volume fraction with surfactant concentration, for all ratios investigated. At constant total surfactant concentration (5, 1, 50 mM), the fitted volume fraction is near constant with a slight maximum at around 70% DDAO that reflects the combination of the CMC decrease with DDAO addition[1], and any packing differences that occur in mixed micelles, discussed in the main paper.

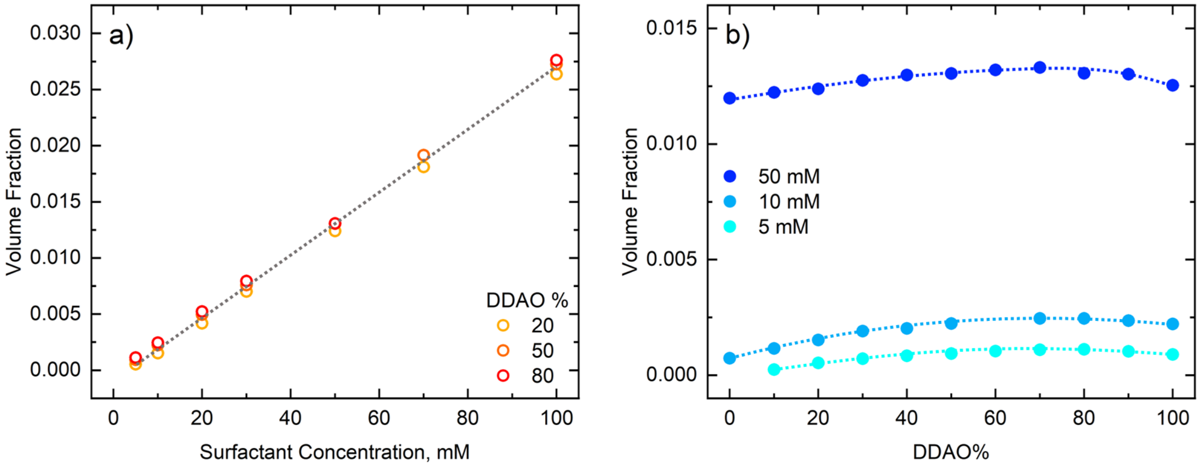

Figure S1. a) Variation in the volume fraction with concentration, obtained by SANS data fitting, for constant SDS:DDAO ratios at 20, 50 and 80% mol DDAO. b) Variation in the volume fraction with surfactant ratio (DDAO%) for constant total surfactant concentrations at 5, 10 and 50 mM. Dashed lines are guides to the eye.

3 SANS data fitting parameters: Backgrounds

The scattering 'background', comprising the D_2O coherent and the surfactant incoherent signal, was extracted by SANS data fitting, and is shown in Fig. S2 (a) for constant surfactant ratio data. The vertical dashed line separates measurements acquired at 5 mm and 2 mm path-length banjo cells (Hellma), corresponding to lower or high concentrations. The horizontal dashed lines indicate the D_2O scattering background, in the limit of no solute present. For a fixed path length, the background increases (approximately linearly) with surfactant concentration, as expected from the incoherent scattering contribution from the hydrogenated surfactants. Fig. S2(b) shows backgrounds obtained at constant total surfactant concentration. There is little variation within the 5-50 mM concentrations, and surfactant ratios, effectively interpreted as within measurement uncertainty. This regular behaviour of the backgrounds is included for completion, and enables a robust approach to SANS data fitting.

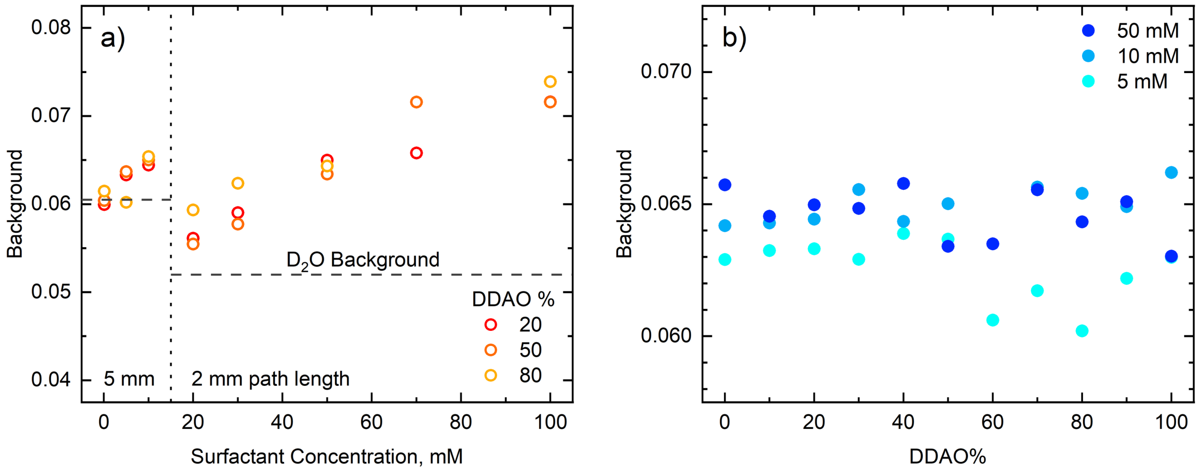

Figure S2. a) Variation in the background with concentration for constant SDS:DDAO ratios at 20, 50 and 80% mol DDAO. The horizontal line segments the data by the Hellma cell path length used in the measured while vertical lines show the solvent, D_2O, background scattering (no solute). b) Variation in the background with surfactant ratio (DDAO%) for constant total surfactant concentrations at 5, 10 and 50 mM.

4 FTIR spectra

Figure S3 shows the baseline corrected, normalised, solvent subtracted and smoothed FTIR spectra for (a) 50 mM and (b) 100 mM total surfactant concentration from 0% to 100% mol DDAO (spectra obtained at 10% DDAO intervals). Absorbance bands used to explore molecular interactions in the main paper are highlighted, namely the anti-symmetric and symmetric CH_2 absorbance and the anti-symmetric SO_3 absorbance region.

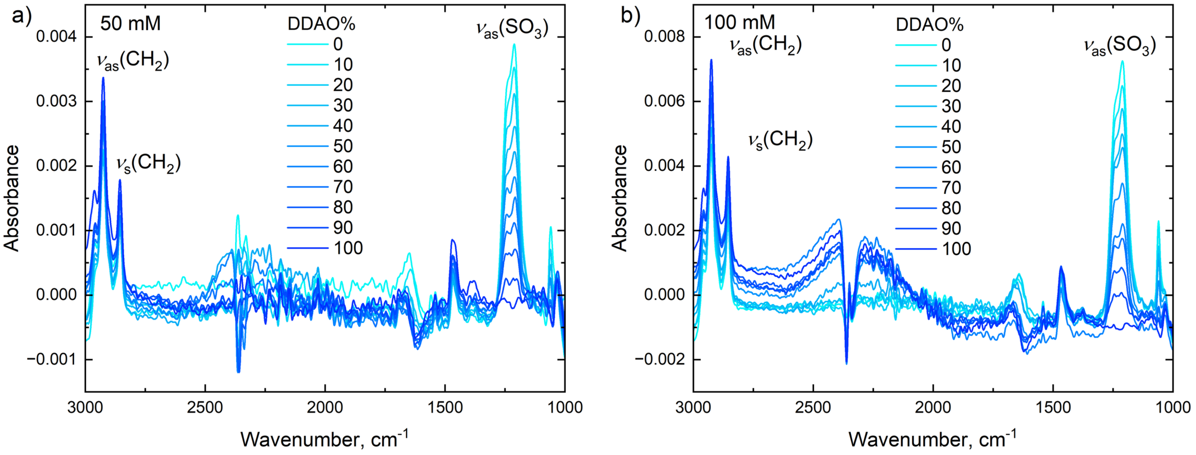

Figure S3. Baseline corrected, normalised, solvent subtracted and finally smoothed FTIR spectra for (a) 50 mM and (b) 100 mM surfactant concentrations from pure SDS (0% DDAO) to pure DDAO (100% DDAO) in 10% concentration steps.

References

1. Tyagi, G. *et al.* Tensiometry and FTIR study of the synergy in mixed SDS:DDAO surfactant solutions at varying pH. *Colloids Surfaces A: Physicochem. Eng. Aspects* **618**, 126414 (2021).
